# Rapid onset type 1 diabetes with anti-PD-1 directed therapy

**DOI:** 10.18632/oncotarget.27665

**Published:** 2020-07-14

**Authors:** Karen Yun, Gregory Daniels, Kathryn Gold, Karen Mccowen, Sandip Pravin Patel

**Affiliations:** ^1^Department of Medicine, University of California San Diego, San Diego, CA 92103, USA; ^2^Division of Hematology-Oncology in the Department of Medicine, University of California San Diego, La Jolla, CA 92093, USA; ^3^Division of Endocrinology in the Department of Medicine, University of California San Diego, La Jolla, CA 92037, USA

**Keywords:** type 1 diabetes, diabetic ketoacidosis (DKA), immune-related adverse event (irAE), immunotherapy, PD-1 inhibitors

## Abstract

Type 1 diabetes is a rare immune-related adverse event (irAE) caused by checkpoint inhibitors with serious risk for diabetic ketoacidosis (DKA). Using our electronic medical record, we identified 1327 adult patients who received PD-(L)1 or CTLA-4 inhibitors from 2013 to 2018. Of the patients who received immunotherapy, 5 (0.38%) patients were found to have type 1 diabetes, all of whom presented with DKA requiring insulin at 20 to 972 days from their first anti-PD-(L)1 dose. All patients were treated with anti-PD-1 therapy (nivolumab or pembrolizumab). Four patients had new onset diabetes with mean HbA1c of 9.1% on DKA presentation and persistent elevations over time. Two patients who tested positive for glutamic acid decarboxylase (GAD) antibodies presented with DKA at 20 and 106 days from first anti-PD-1 administration whereas patients who were autoantibody negative had DKA more than a year later. Type 1 diabetes occurs within a wide time frame after anti-PD-1 initiation and commences with an abrupt course. Our case series suggests that monitoring glycemia in patients on PD-1 inhibitors is not predictive for diabetes occurrence. GAD autoantibodies could portend earlier onset for diabetes, although further prospective studies are needed to elucidate their diagnostic utility and contribution in therapeutic interception.

## INTRODUCTION

Cancer immunotherapy has broadened in clinical use over the last decade with FDA approval for treatment of various malignancies including melanoma, non-small cell lung cancer (NSCLC), renal cell carcinoma, urothelial carcinoma, head and neck carcinomas, cutaneous squamous cell cancer, microsatellite unstable tumors, and Hodgkin’s lymphoma [1, 2]. Programmed cell death 1 (PD-1) (cemiplimab, nivolumab, pembrolizumab) and programmed cell death ligand 1 (PD-L1) (atezolizumab, avelumab, durvalumab) inhibitors promote antitumor activity by downregulating the signals that inactivate T-cell response against tumors [3]. PD-(L)1 inhibitors have shown promise in cancer treatment with clinical trials showing survival benefit for these agents in multiple cancers [1]. However, due to their attenuation of immunologic homeostasis, PD-(L)1 inhibitors can cause dysregulation in self-tolerance, leading to autoimmune toxicities or immune-related adverse events (irAE) in different organ systems [4].

IrAEs include a spectrum of disease states from colitis, pneumonitis, and hepatitis to endocrinopathies such as hypophysitis, thyroiditis and adrenal insufficiency [1]. Insulin-dependent diabetes is a rare irAE with a reported incidence of 0.2% [5]. Diabetic ketoacidosis (DKA) is commonly the initial presentation requiring immediate medical treatment with insulin and close outpatient follow-up. Type 1 diabetes in the general population may be classified as autoimmune or idiopathic. Autoimmune type 1 diabetes is generally associated with positive autoantibodies to islet proteins including glutamic acid decarboxylase (GAD), insulin (IA), insulinoma-associated antigen-2, zinc transporter 8 and islet cells (ICA) [6]. Idiopathic type 1 diabetes may still be autoimmune in nature but those serum markers are absent. Checkpoint inhibitor-induced diabetes has been reported to occur both with and without islet antibodies [7].

Currently, there is insufficient evidence to suggest any reliable biomarker that accurately predicts which patients will develop type 1 diabetes. Case reports and series have described autoantibodies and human leukocyte antigen (HLA) haplotypes in patients who develop type 1 diabetes [8, 9]. However, only a subset of patients who acquire type 1 diabetes are found to have autoantibodies and specific HLA alleles, making these biomarkers poor predictors of diabetes incidence. Given the rarity of type 1 diabetes as an irAE, we sought to characterize the real world diagnosis, management, and sequelae of patients who developed this irAE in the context of their immune checkpoint blockade.

We highlight here the rapid kinetics of type 1 diabetes in patients on checkpoint inhibitors. Type 1 diabetes presented as DKA for all patients in this series and all but one patient had a new diagnosis of diabetes, without antecedent laboratory or imaging findings.

## RESULTS

Type 1 diabetes presenting as DKA in patients on checkpoint inhibitors had a rare incidence of 0.38% in our study. Patients and clinical course are described in Table 1. All five patients with type 1 diabetes had metastatic disease with primary malignancies that included melanoma (*n* = 2), squamous cell lung carcinoma (*n* = 2) and lung adenocarcinoma (*n* = 1) and received therapy with PD-1 inhibitors (nivolumab or pembrolizumab) prior to developing type 1 diabetes. Two patients were on PD-1 inhibitors and ipilimumab, a cytotoxic T-lymphocyte-associated antigen-4 (CTLA-4) inhibitor. For those patients who received dual PD-1 and CTLA-4 inhibitors, one had melanoma and one had NSCLC.

**Table 1:** Clinical history and laboratory data

Patient	Age/Sex	Diagnosis	PD-(L)1 Immunotherapy	Prior Treatments	HbA1c (%)	Time after Anti-PD1 (days)	Prior Diabetes	Concomitant IRAEs
1	70M	Metastatic melanoma	Pembrolizumab	None	11.9	106	Yes	None
2	67M	Metastatic lung squamous cell carcinoma	Nivolumab	Gemcitabine Carboplatin Docetaxel Ipilimumab	9.2	388	No	None
3	49M	Metastatic lung adenocarcinoma	Nivolumab	Carboplatin Paclitaxel Bevacizumab	8.9	20	No	Encephalitis Pneumonitis Myocarditis
4	27M	Metastatic melanoma	Nivolumab	Ipilimumab	6.2	972	No	Adrenal insufficiency Transaminitis
5	74F	Metastatic lung squamous cell carcinoma	Nivolumab	Gemcitabine Carboplatin	9.3	620	No	Hypothyroidism

Time from the first cycle of immunotherapy to subsequent DKA occurrence varied from 20 to 972 days. Four out of the five patients had newly diagnosed diabetes. One patient had a former diagnosis of type 2 diabetes that was poorly controlled due to medication noncompliance and later diagnosed with immunotherapy-induced type 1 diabetes after testing positive for autoantibodies. Apart from this patient, the other patients did not have evidence of persistent hyperglycemia before DKA. Initial labs at DKA presentation for all patients are detailed in Table 2. Labs were notable for mean blood glucose of 668 mg/dL, mean pH on venous blood gas of 7.14 and mean bicarbonate level of 10 mmol/L. Of the four patients with new diabetes, three had high HbA1c levels at the time of DKA with a mean HbA1c of 9.1% and persistent elevations in HbA1c after their DKA event above 7.5%. For one patient with no prior diabetes, HbA1c during DKA admission was 6.2% with mean HbA1c of 7.7% on outpatient follow-up.

**Table 2:** Laboratory findings at the time of DKA

Patient	Glucose (mg/dL)	pH	Anion Gap (mEq/L)	HCO3 (mmol/L)	Beta-Hydroxybutyrate (mg/dL)	Urine Ketones
1	390	7.38	22	18	10.3	Trace
2	971	6.99	38	6	> 83	Negative
3	773	6.93	40	2	> 83	2+
4	314	7.17	32	13	68.8	Negative
5	896	7.23	32	11	> 83	Trace

Testing for biomarkers of type 1 diabetes during or after DKA onset is summarized in Table 3. Two patients were GAD antibody positive including the patient with previous type 2 diabetes who was both GAD antibody and IA positive. All patients were negative for ICA. HLA-A2 was negative in the one patient who underwent HLA testing. Serum c-peptide at or around the time of DKA was undetectable or low in all patients except for one patient with newly diagnosed diabetes whose serum c-peptide was measured 4 months after DKA and undetectable at that time.

**Table 3:** Autoantibodies and HLA data

Patient	GAD	ICA	IA	HLA
1	+	-	+	N/A
2	-	-	N/A	N/A
3	+	-	-	N/A
4	-	N/A	N/A	N/A
5	-	-	N/A	-^1^

^1^HLA-A2 negative.

Additionally, serum amylase and lipase levels, for which one of these is tested for with every immunotherapy dose, were not consistently associated with antecedent pancreatitis in cases of type 1 diabetes. All patients had normal (*n* = 3) or modest elevations (*n* = 2) in serum amylase less than two times the upper limits of normal after anti-PD-1 initiation. Results for lipase showed similar trends to amylase with the exception of one patient who had a serum lipase greater than three times the upper limits of normal. Along with laboratory data, abdominal CT scans, done every 2–3 months as part of standard of care response assessment, did not reveal any imaging findings of pancreatitis precluding the development of type 1 diabetes.

Besides type 1 diabetes, three patients encountered other irAEs. One patient had concurrent encephalitis, pneumonitis and myocarditis following two cycles of nivolumab. A second patient had adrenal insufficiency and transaminitis on both nivolumab and ipilimumab while a third patient had hypothyroidism on nivolumab alone. For the last two patients, these immune-related diseases appeared before the onset of diabetes. No new endocrinopathies or acute exacerbations of chronic endocrine disorders were observed during DKA - thyroid-stimulating hormone, free thyroxine, and cortisol levels were normal in all patients.

Patients admitted for DKA sought care at the hospital for a variety of symptoms that included fatigue, shortness of breath, confusion, blurry vision and weight loss. All patients endorsed polyuria. Polydipsia (*n* = 4), abdominal pain (*n* = 4), nausea (*n* = 3), and emesis (*n* = 3) were among other commonly reported symptoms.

Intravenous fluids and insulin are critical for the management of DKA with subcutaneous insulin as the mainstay outpatient treatment for type 1 diabetes. Insulin requirements on discharge and follow-up varied among patients in our case series. Two patients with additional irAEs received high dose prednisone on discharge and required 0.29 to 0.85 units/kg/day of insulin while the patient with adrenal insufficiency on chronic hydrocortisone required 0.4 units/kg/day of insulin. One patient discharged without glucocorticoids required 1.22 units/kg/day of insulin. On follow-up, there was evidence of a honeymoon phase at 4 months after DKA onset in the patient with prior diabetes where the insulin requirement decreased from 0.87 to 0.51 units/kg/day, suggesting some endogenous insulin secretion by residual beta cells. For the remaining patients, it is less certain whether or not they underwent a honeymoon period given the confounding effect of glucocorticoid use either at discharge or at 6-month follow-up.

Upon type 1 diabetes diagnosis, continuation of therapy with PD-1 inhibitor was mixed amongst patients as shown in Table 4. In the patient with previously uncontrolled type 2 diabetes and metastatic melanoma, pembrolizumab was continued for an additional 10 weeks with progression of disease at 16 weeks after DKA. Clinical course continued to be complicated by poorly controlled diabetes in the setting of poor compliance leading to multiple readmissions for symptomatic hyperglycemia as well as hypoglycemia. Another patient with metastatic melanoma who completed his last dose of nivolumab 1.6 weeks prior to DKA had no progression of disease at one year from last immunotherapy administration and no readmissions related to diabetes. However, labs were notable for recurrent episodes of hyperglycemia due to adjustments in outpatient glucocorticoid dosage. One patient with refractory NSCLC switched from nivolumab to atezolizumab after immunotherapy was held for 9 months following diabetes onset and remained on atezolizumab for 53.1 weeks. Here, atezolizumab demonstrated clinical benefit in terms of survival despite disease progression. Another patient with NSCLC who received ipilimumab and nivolumab for 20.6 additional weeks was determined to have progression of disease at 21 weeks after DKA. In the third patient with NSCLC, clinical benefit was observed on nivolumab but further immunotherapy was held after initial DKA occurrence with progression of disease at 64 weeks. All patients with NSCLC overall had difficult to control type 1 diabetes with readmissions for DKA. Precipitants for recurrent DKA were multifactorial including poor medical compliance, glucocorticoids and acute medical conditions.

**Table 4:** Clinical course after DKA

Patient	Anti-PD1 Therapy	Duration of Immunotherapy (weeks)	Progression of Disease	Time to Progression (weeks)
1	Continued	10	Yes	16
2	Continued	20.6	Yes	21
3	Held^1^	53.1	Yes	N/A^2^
4	Discontinued	0	No	-
5	Discontinued	0	Yes	64

^1^Patient was switched from nivolumab to atezolizumab after immunotherapy was held for 9 months following DKA occurrence. ^2^Patient with disease progression prior to DKA.

## DISCUSSION

Type 1 diabetes is a rare but serious side effect of immune checkpoint inhibitors. Multiple case reports have described fulminant diabetes in patients on PD-1 inhibitors characterized by sudden onset ketoacidosis, severe hyperglycemia and close to normal HbA1c of less than 8.5% on initial presentation [10–14]. Additional features of fulminant diabetes may include elevated serum pancreatic enzymes, negative islet-related autoantibodies and low fasting serum c-peptide levels [15]. Fulminant diabetes is a subclass of diabetes recognized in Japan as different from classic autoimmune type 1 diabetes in that the mechanism of fulminant diabetes is thought to be pancreatic β-cell destruction in the absence of autoantibodies [15, 16]. In our case series, only one patient met the criteria for fulminant diabetes as the other patients had elevated HbA1c values. Nonetheless, patients with newly diagnosed diabetes in our study developed type 1 diabetes with abrupt onset in the setting of acute hyperglycemia which suggests that diabetes, regardless of fulminant status, occurs with a rapid course in patients on PD-1 inhibitors.

Autoreactive T cells are the main mediators in pancreatic beta-cell destruction while autoantibodies serve as markers of autoimmune diabetes [17]. Islet cell autoantibodies have been detected in about half of published case reports and their roles in type 1 diabetes in relation to immunotherapy have yet to be fully elucidated. Here, two patients who were GAD antibody positive developed type 1 diabetes at 20 days and 106 days from anti-PD-1 initiation compared to more than one year in GAD antibody negative patients. This is consistent with previous observations that GAD positivity may be associated with a shorter time between anti-PD-1 administration to type 1 diabetes onset [14, 18, 19]. A literature review by Gauci *et al*. found that the median time to DKA was 3 weeks in patients on PD-1 inhibitors with positive GAD antibody [19]. Several studies of patients with type 2 diabetes have shown the presence of GAD antibody to be associated with progression to insulin dependence in 40–84%, although the humoral markers of type 1 diabetes are not known to be pathogenic [20–22].

Predicting who will develop type 1 diabetes on anti-PD-1 therapy is a challenge and a few case reports recommend surveillance of blood glucose [12, 14, 19]. However, a review of glycemia in our case series did not show any sustained elevations in blood glucose levels preceding newly diagnosed diabetes. Our findings are consistent with the results of a retrospective study done by Magis *et al*. that looked at the fasting glucose of patients before, during and after anti-PD-1 treatment and found that all cases of type 1 diabetes occurred in patients with normoglycemia prior to DKA occurrence [23]. In the setting of these findings, Magis *et al*. advises against monitoring of blood glucose in patients on immunotherapy [23]. Patients in our case series had their blood glucose checked as part of the basic metabolic panel to assess glycemia, renal function and other electrolytes while on immunotherapy. In the absence of symptoms related to hyperglycemia, routinely checking blood glucose is unlikely to lead to an early diagnosis of DKA.

Regarding HbA1c, there is insufficient data to suggest its utility in estimating diabetes risk. Indeed, trending HgbA1c likely has no value in approximating the incidence of fulminant diabetes. For acute type 1 diabetes, it is less certain. Our data are limited as the HbA1c levels for several patients prior to diabetes diagnoses are unknown. Nevertheless, in one of our patients, HbA1c about 2.5 months prior was 5.6% and then 9.3% at the time of DKA. Like blood glucose, following HbA1c levels may not allow early diagnosis of diabetes. Stamatouli *et al*. suggests that elevated HbA1c levels at the time of diabetes diagnosis potentially signifies hyperglycemia over a short interval of time, underscoring the rapid progression of type 1 diabetes [24].

Once type 1 diabetes occurs, patients exhibit elevated HbA1c values associated with labile blood glucose measurements and multiple hyperglycemic episodes. Unlike other irAEs where glucocorticoids are the mainstay of therapy, type 1 diabetes is primarily treated with insulin. Glucocorticoids are not typically used for the management of immunotherapy-induced type 1 diabetes due to their propensity to cause hyperglycemia [8]. However, high dose prednisone was used in two patients in our study with other irAEs without evidence of benefit for diabetes.

A number of irAEs such as myositis and pneumonitis resolve with glucocorticoids while some autoimmune endocrinopathies result in permanent organ damage [25–28]. Chronic insulin dependence indicates the irreversible nature of diabetes in patients on immune checkpoint inhibitors. Developing type 1 diabetes secondary to immunotherapy poses a risk for recurrent hyperglycemia and DKA such that once patients lose endocrine pancreatic function and are insulin dependent, they may continue immunotherapy for lethal malignancies, as pancreatic damage is likely irreparable. As of 2018, the American Society of Clinical Oncology (ASCO) practice guidelines in collaboration with the National Comprehensive Cancer Network (NCCN) recommend holding immunotherapy upon type 1 diabetes or DKA diagnosis until glucose control is achieved [29]. Resumption of anti-PD1 therapy differed among patients in our case series and all patients who remained on immunotherapy after DKA had progression of disease, necessitating additional studies to clarify the clinical benefit of resuming checkpoint inhibitors in patients with irAEs.

## MATERIALS AND METHODS

Using our electronic medical record under an IRB-approved protocol, we identified 1327 patients who received immunotherapy with PD-(L)1 inhibitors (atezolizumab, avelumab, durvalumab, nivolumab, pembrolizumab) or ipilimumab from 2013 to 2018. Our search criteria included patients with all tumor types including solid tumors and hematologic malignancies as well as patients enrolled in clinical trials with immunotherapy. Additional inclusion criteria of blood glucose greater than 200 mg/dL and bicarbonate less than 18 mmol/L were used to abstract patients for further chart review and identify those who developed DKA in the setting of immunotherapy-induced type I diabetes.

## CONCLUSIONS

Our case series illustrates the rare incidence of immunotherapy-induced type 1 diabetes and describes the rapid course of this disease in patients. In our case series, the development of DKA relative to initial dosing of immune checkpoint blockade had a wide range from 20 to 972 days. Long-term insulin was the primary therapeutic modality for diabetes, reflecting permanent loss in endocrine pancreatic function. Continuation of anti-PD-1 therapy varied among patients in our case series with progression of disease in all patients who resumed immunotherapy. Regardless of whether or not patients remain on checkpoint inhibitors, those with immunotherapy-induced diabetes are at risk for hyperglycemia and recurrent DKA. Surveillance of glycemia or HbA1c does not predict diabetes but does have a role after type 1 diabetes arises as glycemia fluctuates and elevated HbA1c levels persists. Furthermore, GAD antibodies are present in about half of patients who develop type 1 diabetes after immunotherapy, warranting additional investigations into whether this is all association and a marker of immune attack. Given the absence of prescient laboratory or imaging findings in patients who develop type 1 diabetes on anti-PD-1 therapy, patients should be counseled on the symptoms of hyperglycemia which includes polyuria, polydipsia, abdominal pain, nausea and emesis and seek medical attention immediately.

## Abbreviations

ASCO: American Society of Clinical Oncology
DKA: diabetic ketoacidosis
GAD: glutamic acid decarboxylase
IA: insulin antibody
ICA: islet cell antibody
irAE: immune-related adverse event
NCCN: National Comprehensive Cancer Network
NSCLC: non-small cell lung cancer
PD-1: programmed death 1
PD-L1: programmed death ligand 1
